# Palaeognaths Reveal Evolutionary Ancestry of the Avian Major Histocompatibility Complex Class II

**DOI:** 10.1093/gbe/evae211

**Published:** 2024-10-03

**Authors:** Piotr Minias, Wiesław Babik

**Affiliations:** Faculty of Biology and Environmental Protection, Department of Biodiversity Studies and Bioeducation, University of Lodz, Banacha 1/3, 90-237 Lodz, Poland; Institute of Environmental Sciences, Faculty of Biology, Jagiellonian University, Kraków, Poland

**Keywords:** birds, evolution, MHC, orthology, phylogenetics, reptiles

## Abstract

The multigene family of the major histocompatibility complex (MHC) codes for the key antigen-presenting molecules of the vertebrate immune system. In birds, duplicated MHC class II (MHC-II) genes are highly homogenized by concerted evolution, and thus, identification of their orthologous relationships across long evolutionary timescales remains challenging. Relatively low evolutionary rate of avian MHC class IIA genes has been expected to provide a promising avenue to allow such inferences, but availability of MHC-IIA sequences in nonmodel bird species has been limited until recently. Here, taking advantage from accumulating genomic resources, we identified and analyzed MHC-IIA sequences from the most basal lineage of extant birds (Palaeognathae). Conserved region of the MHC-IIA membrane-proximal domain was used to search for orthologous relationships between palaeognath birds and nonavian reptiles. First, analyses of palaeognath sequences revealed the presence of a separate MHC-IIA gene lineage (*DAA3*) in kiwis, which did not cluster with previously described avian MHC-IIA lineages (*DAA1* and *DAA2*). Next, phylogenetic reconstruction showed that kiwi *DAA3* sequences form a single well-supported cluster with turtle MHC-IIA. High similarity of these sequences most likely reflects their remarkable evolutionary conservation and retention of ancient orthologous relationships, which can be traced back to basal archosauromorphs ca. 250 million years ago. Our analyses offer novel insights into macroevolutionary history of the MHC and reinforce the view that rapid accumulation of high-quality genome assemblies across divergent nonmodel species can substantially advance our understanding of gene evolution.

**Significance**

In this study, we took advantage from accumulating genomic resources to extract MHC-IIA sequences from the most basal lineage of extant birds (Palaeognathae) and search for their orthologous relationships across long evolutionary timescales. Phylogenetic analyses revealed separate MHC-IIA gene lineage (*DAA3*) in kiwis, which failed to cluster with previously described avian MHC-IIA lineages (*DAA1* and *DAA2*). Instead, *DAA3* formed a single well-supported cluster with turtle MHC-IIA sequences. We conclude that high similarity of these sequences most likely reflects their remarkable evolutionary conservation and retention of ancient orthologous relationships, which can be traced back to basal archosauromorphs ca. 250 million years ago.

## Introduction

Proteins of the major histocompatibility complex (MHC) are a key component of adaptive immunity, as they present antigenic peptides to T-cells, which initiates the adaptive immune response (Murphy et al. 2022). MHC proteins are encoded by the most polymorphic genes in vertebrate genomes (Klitz et al. 2012), and their polymorphism is mainly maintained by the evolutionary arms race with pathogens, leading to balancing selection that likely operates through multiple, still incompletely understood, mechanisms (Radwan et al. 2020). MHC genes are not only characterized by extraordinary polymorphism, but in many taxa, they also form dynamically evolving multigene families, experiencing frequent gene duplications and losses (Minias et al. 2019; He et al. 2022), with duplicated gene copies undergoing both divergent and concerted evolution (Nei and Rooney 2005). The two main MHC classes (class I, MHC-I, and class II, MHC-II) differ both in the protein structure and in the origin of the antigenic peptides they present (mostly intracellular for MHC-I and extracellular for MHC-II; Pishesha et al. 2022). Genes encoding each MHC class can also exhibit distinct evolutionary dynamics (Hughes and Nei 1989; 1990).

Functional MHC-II molecules have a form of heterodimer consisting of α and β units encoded by distinct IIA and IIB genes. Mammals have several MHC-II proteins, such as DP, DQ, or DR, encoded by pairs of tightly linked IIA and IIB genes, whose orthologous relationships have been identified between eutherian orders that diverged around 80 to 100 million years ago (mya) (Hughes and Nei 1990). In birds, the identification of orthologous MHC-II genes between orders has been challenging because concerted evolution through interlocus recombination and gene conversion often leads to relative homogenization of sequences between different MHC-II genes, as seen for example in passerine birds (Edwards et al. 1995; Zagalska-Neubauer et al. 2010; Westerdahl et al. 2022). However, studies in owls (Burri et al. 2008; 2010), followed by large-scale comparative studies of avian MHC-II genes (Goebel et al. 2017; Minias et al. 2023) revealed the presence of two major MHC-II gene lineages, derived from a duplication that predates the origin of extant birds ca. 90 to 110 mya (Jarvis et al. 2014; Yonezawa et al. 2017; Stiller et al. 2024). In fact, orthologs of both lineages can be identified even between distantly related bird orders. Each lineage is represented by both IIA and IIB genes, giving rise to *DAA1/DAB1* and *DAA2/DAB2* pairs. Both lineages have been retained in many avian orders, but losses of each lineage have also been inferred (Minias et al. 2023). Three observations strongly suggest intense coevolution between IIA and IIB genes within each lineage, although direct evidence is still lacking. First, there is perfect correspondence between the retention and loss of IIA and IIB from a given lineage in the phylogeny (Minias et al. 2023). Second, sequence divergence between lineages is limited to about 30 amino acids at the 5′-end of the exon 3 of IIA and IIB genes—the very region involved in interactions between the α and β chains of the MHC-II dimer (Burri et al. 2008; 2010; Goebel et al. 2017; Minias et al. 2023). The remaining parts of the genes have apparently been homogenized by interlocus exchange of sequences, which generally cluster by species rather than by gene. Third, positive selection following the origin of the two lineages fixed radical amino acid substitutions within the membrane-proximal domain (encoded by exon 3) of both *DAA* and *DAB* genes (Burri et al. 2010; Minias et al. 2023).

Although previous research has shed some light on the evolution of the divergent MHC-II gene lineages in birds, it has not led to an understanding of their evolutionary origin. So far, evolutionary history of the two avian MHC-II lineages has been traced back to the duplication event preceding the radiation of extant birds, but it has been proposed that their orthology may possibly be tracked over even longer timescales (Goebel et al. 2017). Specifically, relatively low evolutionary rate of MHC-IIA genes has been expected to provide a promising avenue for an effective identification of ancient orthologies and allow inferences on the evolutionary origins of the avian MHC-II (Goebel et al. 2017). However, availability of MHC-IIA sequences in nonmodel avian species has long been limited, and only recently, a rapid accumulation of genomic data promoted effective macroevolutionary analyses of MHC-IIA sequences in birds (Minias et al. 2023). Here, we extracted MHC-IIA exon 3 sequences from the available genome assemblies of palaeognath birds to investigate the evolution of MHC-IIA genes at a deeper evolutionary perspective. As palaeognaths form the most basal lineage of extant birds, which originated in Early–Late Cretaceous boundary period around 90 to 110 mya (Jarvis et al. 2014; Yonezawa et al. 2017; Stiller et al. 2024), we expected that the conserved regions of their MHC-IIA genes may still retain the signature of orthology with MHC-IIA genes of related amniote lineages, such as nonavian reptiles. To explore this issue, we investigated phylogenetic relationships of MHC-IIA among palaeognath birds and three major evolutionary lineages of extant reptiles, including squamates, turtles, and crocodilians. In general, all extant birds and nonavian reptiles are classified within a single clade of Diapsida, but diapsid phylogeny is still not fully resolved (Shedlock and Edwards 2009). One of the leading hypotheses poses that diapsids diverged in the Middle or Late Permian around 250 to 270 mya into two major evolutionary lineages of lepidosauromorphs (squamates and sphenodontians) and archosauromorphs, which have diverged rapidly around the Permian–Triassic boundary (ca. 250 mya) into the lineages leading to extant turtles, crocodilians, and birds (Simões et al. 2018, Colston et al. 2020, Martínez et al. 2021). Hence, we hypothesized that avian (palaeognath) MHC-IIA is more likely to retain signature of orthology with either turtles or crocodilians, rather than squamate reptiles.

## Materials and Methods

### Phylogeny of Palaeognath MHC-IIA

We used 23 genome assemblies of extant palaeognath species available in the Genome NCBI database (as accessed in January 2024) to extract MHC-IIA membrane-proximal α2 domain (exon 3) sequences. For this purpose, we generated a full-length (282 bp) consensus MHC-IIA exon 3 query from palaeognath sequences used in previous research (Minias et al. 2023). BLAST searches were conducted with *blastn* algorithm designed to search for relatively short queries of moderate similarity in a cross-species framework (default settings: reward for matching bases: 2, penalty for mismatching bases: −3, cost to create a gap in alignment: 5, cost to extend gap in an alignment: 2). In total, we retrieved 43 MHC-IIA exon 3 sequences (including 38 full-length sequences) from 19 species representing all five palaeognath orders, i.e. Apterygiformes, Casuariiformes, Rheiformes, Struthioniformes, and Tinamiformes (supplementary table S1, Supplementary Material online). All sequences were aligned using two approaches: (i) MUSCLE algorithm (Edgar 2004) implemented in Geneious v.10.0.5 software (Biomatters Ltd., Auckland, New Zealand) and (ii) MACSE v.2 software (Ranwez et al. 2018). Both approaches (default settings) produced identical alignments with no gaps, frameshifts, or stop codons, so no manual editing was required.

Phylogenetic reconstruction was based on the short (90 nt) upstream (5′) fragment of MHC-IIA exon 3 (corresponding to 30 *N*-terminal amino acids of the exon), which was shown to yield the highest power to resolve orthology of MHC-IIA genes in birds (Minias et al. 2023). Sequences showing high similarity within species (>95% of pairwise nucleotide identity in the upstream region) were removed (*n* = 14 out of 43), retaining 29 sequences for the analysis. Known *DAA1* and *DAA2* owl (Strigiformes) sequences were added to the alignment as a reference (Blakiston's fish owl *Bubo blakistoni*, genome assembly GenBank no. GCA_004320225, contig GenBank nos. BJCB01040766 and BJCB01033660 for *DAA1* and *DAA2*, respectively), while the Chinese giant salamander *Andrias davidianus* was used as outgroup (Genbank no. KF611869).

Phylogenetic reconstruction was conducted using Bayesian tree-building algorithms, as implemented in MrBayes v.3.2.6 (Ronquist et al. 2012). Amino acid sequences were used for the clustering. Substitution models were compared in Mega v.6.0 software (Tamura et al. 2013) using maximum likelihood approach, and the best model was selected based on the lowest Bayesian information criterion. We used two independent chains in the analysis, each consisting of one million iterations, burin-in length of 250,000 steps, and sampling every 500th tree, retaining 1,500 expected trees per chain. All the analyses were run using the molecular clock with uniform branch lengths models and the default uninformative flat priors. Models with unconstrained and exponential branch lengths were also tested, but they provided qualitatively similar tree topologies (results not shown). Convergence of the two independent chains was confirmed by the average standard deviation of split frequencies approaching zero (Lakner et al. 2008) and the potential scale reduction factors approaching one (Gelman and Rubin 1992). Geneious v.10.0.5 was used to determine extended majority rule consensus tree topology.

### Phylogeny of Palaeognath and Reptilian MHC-IIA

Since phylogenetic analysis of palaeognath sequences revealed three distinct clusters of sequences, likely corresponding to three gene lineages (*DAA1*-*DAA3*; see Results for details), we prepared consensus query sequences (upstream 90 nt region of MHC-IIA exon 3) within each gene lineage separately for different palaeognath orders. In total, we generated seven queries, including one *DAA3* (Apterygiformes), four *DAA2* (Apterygiformes, Rheiformes, Struthioniformes, and Tinamiformes), and two *DAA1* (Casuariiformes and Tinamiformes) sequences. All queries were blasted with *blastn* algorithm against the Nucleotide NCBI database (GenBank, EMBL, DDBJ, PDB, and RefSeq). Three best-matching MHC-IIA exon 3 sequences of nonavian reptiles were retrieved from each BLAST search, and sequences overlapping between different searches were removed. In total, 15 unique reptilian sequences were retained for the analysis, representing three major evolutionary lineages of nonavian reptiles, i.e. Squamata, Testudines, and Crocodylia (supplementary table S2, Supplementary Material online). All these sequences were aligned with palaeognath MHC-IIA sequences, including all sequences available for Apterygiformes and consensus sequences for other palaeognath orders (separately for *DAA1* and *DAA2*). We used the two aligning algorithms described above (MUSCLE and MACSE), both producing identical alignments. Pairwise nucleotide similarity (% identity) matrix was extracted from the alignment using Geneious v.10.0.5. Mean ± SE nucleotide similarity values are reported.

Next, we performed phylogenetic analysis of palaeognath *DAA1*-*DAA3* sequences together with the retrieved reptilian sequences. This was first conducted for the upstream 90 nt region of MHC-IIA exon 3, which was expected to yield the highest power to resolve orthology. Second, to check whether the obtained relationships could have been caused by convergence, we reran these analyses for third codon positions, as they are mostly unaffected by selection and expected to better reflect phylogenetic relationships. Under the convergence scenario, phylogenetic relationships inferred from third codon positions and full codons should differ, reflecting neutral gene history and functional variation that evolved under convergent selection, respectively (Burri et al. 2010). Finally, to check if the signal of orthology is retained along the entire exon 3 sequence, the analyses were rerun for the remaining downstream region (192 nt). Struthioniformes *DAA2* was excluded from the latter analysis, as it had incomplete downstream region. All phylogenetic analyses of palaeognath and reptilian sequences were conducted in MrBayes v.3.2.6 software following the same algorithm as used for palaeognath-only sequences.

As our phylogenetic reconstruction provided evidence for the retention of orthology between Apterygiformes *DAA3* and Testudines, but not Crocodylia (see Results for details), we performed additional BLAST searches across all crocodilian genomes (*n* = 6 species) available in the Genome NCBI database. Specifically, we aimed to retrieve any crocodilian MHC-IIA sequences with high nucleotide similarity to *DAA3*, which could have been missed by previous BLAST searches. For this purpose, we used the same *DAA3* query and *blastn* algorithm, as described previously. Only three sequences (one per species) were retrieved from *Alligator mississippiensis* (JAGPOW010002857.1), *Caiman crocodilus* (NC_081834.1), and *Crocodylus rhombifer* (JAVSML010016059.1).

### Selection Inference

Due to striking similarity of MHC-IIA sequences between Apterygiformes (*DAA3*) and Testudines (see Results for details), selection inference focused on these two clades. In the analyses, we used two complementary maximum likelihood approaches implemented in the Datamonkey v.2.0 webserver (Weaver et al. 2018). In the first step, we used branch-specific fixed effects likelihood (FEL) approach (Kosakovsky Pond and Frost 2005) to test if the divergence of *DAA2* and *DAA3* gene lineages in Apterygiformes was driven by directional positive selection. For this purpose, the signature of positive selection (*dN*/*dS* > 1) was quantified along at the branch that separated the two gene lineages. Statistical significance of codon-specific positive selection signature was inferred using likelihood ratio tests based on the asymptotic χ^2^ distribution. We also used contrast-FEL (Kosakovsky Pond et al. 2021) to search for codons showing higher *dN*/*dS* ratios between gene lineages (at the branch separating the lineages) than within gene lineages (at the branches separating sequences within lineages). All the analyses were run using default settings and input trees inferred directly from the alignment.

In the second step, we analyzed selection acting between *DAA3* of Apterygiformes and Testudines. For this purpose, we retrieved 145 turtle MHC-IIA exon 3 sequences available in the Nucleotide NCBI database and aligned them in Geneious v.10.0.5. Sequences showing very high similarity within species (>98% of pairwise nucleotide identity) were removed from the alignment, retaining 76 sequences that represented 13 turtle species from 11 genera (*Caretta*, *Chelonia*, *Chelonoidis*, *Chrysemys*, *Dermochelys*, *Gopherus*, *Malaclemys*, *Mauremys*, *Pelodiscus*, *Terrapene*, and *Trachemys*). Branch-specific FEL approach was used to test if the divergence of MHC-IIA between Testudines and Apterygiformes (*DAA3*) was driven by directional positive selection. Contrast-FEL was also used to search for additional codons showing higher *dN*/*dS* at the branches between the two clades compared to branches within the clades.

## Results

**Fig. 1.**
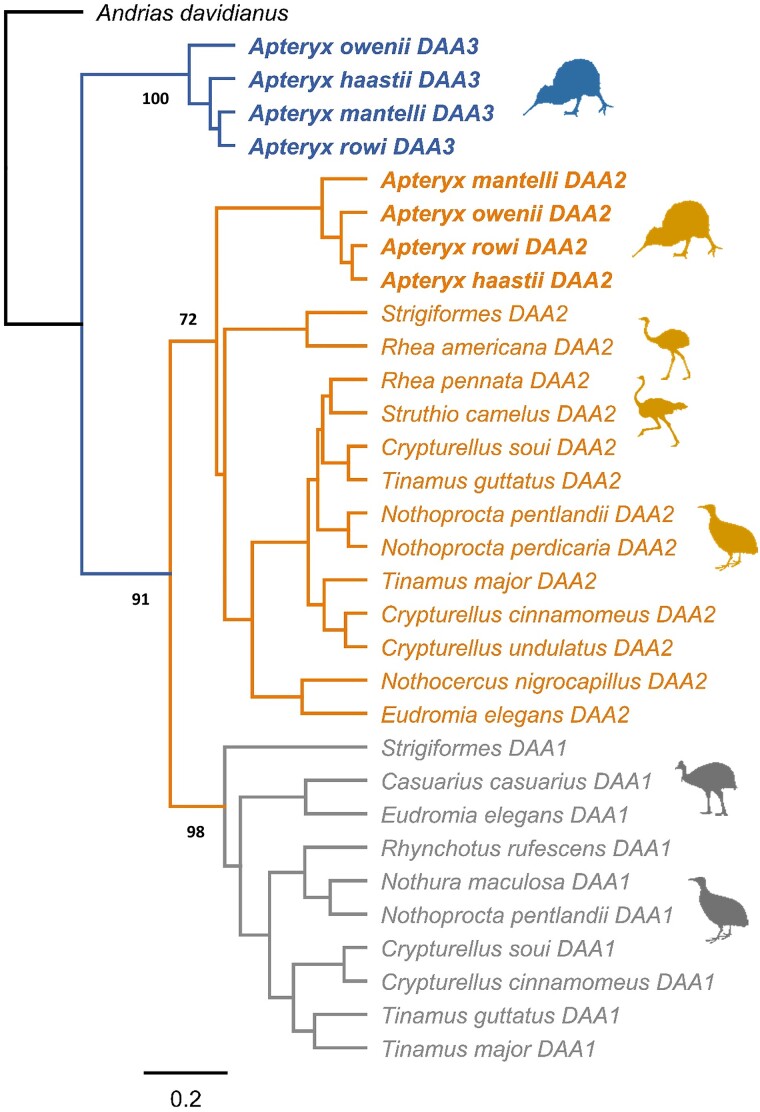
Consensus Bayesian topology of MHC-IIA sequences in Palaeognathae birds. Three MHC-II gene lineages are marked in gray (*DAA1*), yellowish (*DAA2*), and blue (*DAA3*). Kiwi (Apterygiformes) sequences are bolded. Phylogenetic relationships were reconstructed based on the upstream 90 nt (30 aa) region of MHC-IIA exon 3. Bayesian posterior probabilities are provided for major clusters. Owl (Strigiformes) sequences were used as *DAA1* and *DAA2* gene lineage reference (GenBank nos. BJCB01040766 and BJCB01033660; genome assembly GenBank no. GCA_004320225). *Andrias davidianus* was used as outgroup (GenBank no. KF611869). Silhouettes mark different orders of Palaeognathae birds (Apterygiformes, Casuariiformes, Rheiformes, Struthioniformes, and Tinamiformes).

Phylogenetic inference revealed the presence of three distinct, highly supported palaeognath MHC-IIA clusters (Fig. 1). Two clusters represented *DAA1* and *DAA2* gene lineages (Fig. 1), which were previously described in Palaeognathae and have originated through the ancient duplication of ancestral avian MHC-IIA gene (Minias et al. 2023). The third cluster (100% posterior probability) contained sequences from a single palaeognath order (Apterygiformes) and sequences from the same taxa were also found in the *DAA2* cluster, providing evidence for clustering by their apparent orthology rather than by species (Fig. 1). Consequently, the third cluster was inferred to represent a separate MHC-IIA gene lineage (*DAA3*) putatively paralogous to *DAA1* and *DAA2* genes.

**Fig. 2.**
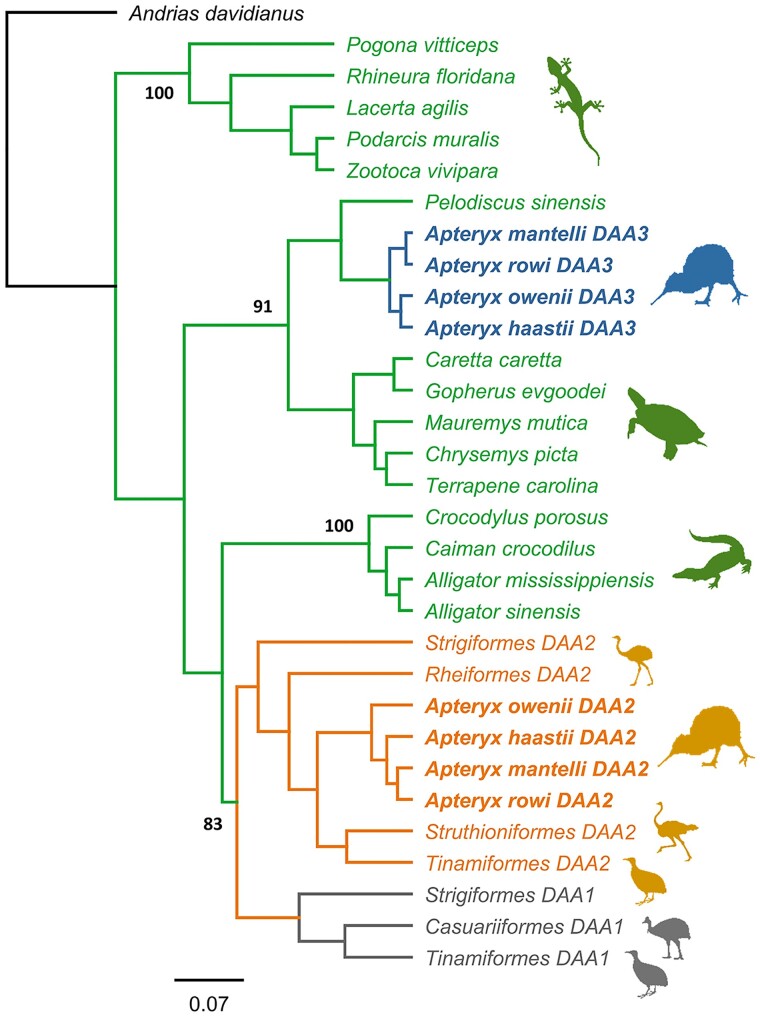
Consensus Bayesian topology of MHC-IIA sequences in Palaeognathae birds and reptiles. Three avian MHC-IIA gene lineages are marked in gray (*DAA1*), yellowish (*DAA2*), and blue (*DAA3*), while reptilian MHC-IIA sequences are marked in green. Kiwi (Apterygiformes) *DAA* sequences are bolded. Phylogenetic relationships were reconstructed based on the upstream 90 nt region of MHC-IIA exon 3. Bayesian posterior probabilities are provided for major clusters. Owl (Strigiformes) sequences were used as *DAA1* and *DAA2* gene lineage reference (GenBank nos. BJCB01040766 and BJCB01033660; genome assembly GenBank no. GCA_004320225). *Andrias davidianus* was used as outgroup (GenBank no. KF611869). Silhouettes mark different orders of Palaeognathae birds (Apterygiformes, Casuariiformes, Rheiformes, Struthioniformes, and Tinamiformes) and reptiles (Crocodylia, Squamata, and Testudines).

Reconstruction of phylogenetic relationships among MHC-IIA of palaeognaths and nonavian reptiles (based on 90 nt at the 5′-end of exon 3, henceforth referred as the upstream region) revealed a single well-supported (91% posterior probability) cluster of Apterygiformes *DAA3* and Testudines sequences (Fig. 2). MHC-IIA sequences from other orders of nonavian reptiles (Crocodylia and Squamata) formed distinct clusters with 100% posterior probability, and palaeognath *DAA1* and *DAA2* gene lineages also formed a distinct cluster (Fig. 2). Consistently with the results of phylogenetic reconstruction, Apterygiformes *DAA3* sequences (upstream region) showed higher nucleotide similarity to Testudines (89.72 ± 0.41 [SE] %) than Crocodylia (81.39 ± 0.31%) or Squamata (76.56 ± 0.25%). Nucleotide similarity of palaeognath *DAA1*/*DAA2* sequences to nonavian reptiles was relatively low (Testudines: 82.03 ± 0.37%, Crocodylia: 81.34 ± 0.79%, and Squamata: 81.34 ± 0.79%). Additional BLAST searches across available crocodilian genomes revealed no MHC-IIA sequences with high nucleotide similarity to Apterygiformes *DAA3* (mean similarity: 81.64 ± 0.75%), providing no support for the retention of orthologs in both lineages.

The analysis of third codon positions (upstream region) did not provide any clear evidence for different tree topology compared to full codon analysis (supplementary fig. S1, Supplementary Material online). Hence, the scenario of functional sequence convergence was not supported, although most major clusters received low support (supplementary fig. S1, Supplementary Material online), likely reflecting low power of short (30 nt) sequences to resolve phylogenetic relationships. The clustering pattern revealed by the analysis of the upstream region was not retained when downstream region of MHC-IIA exon 3 was analyzed. Here, Apterygiformes *DAA3* and *DAA2* were still separated, but no signal of orthology was retained between Apterygiformes and Testudines, since sequences clustered as expected based on species relatedness (supplementary fig. S2, Supplementary Material online).

**Fig. 3.**
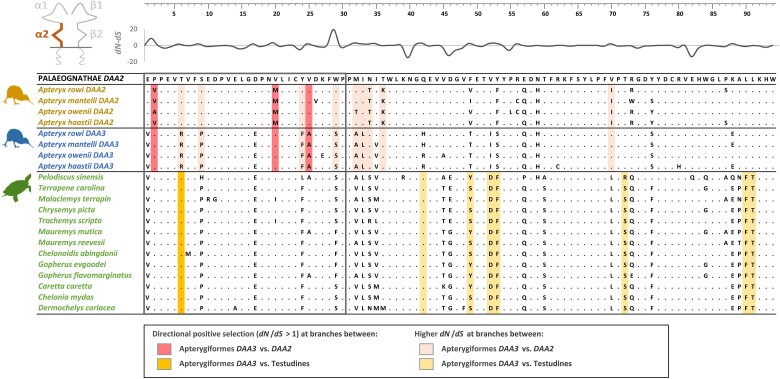
Alignments of amino acid sequences of MHC class IIA exon 3 (α2 domain) in Apterygiformes (kiwi) and Testudines (turtles). Apterygiformes *DAA3* and *DAA2* gene lineages were shown separately. Dots indicate amino acids identical with the reference consensus palaeognath *DAA2* sequence. FEL approach was used to test for significant signature of directional positive selection (*dN*/*dS* > 1) at branches between (i) Apterygiformes *DAA3* versus *DAA2* and *ii*) Apterygiformes *DAA3* versus Testudines. Contrast-FEL was used to test for higher *dN*/*dS* at branches between than within gene lineages (Apterygiformes *DAA2* vs. *DAA3*) or clades (Apterygiformes *DAA3* vs. Testudines). Codons identified by FEL and contrast-FEL are marked with dark and light colors, respectively (according to the legend). Variation in selection parameter (*dN–dS*) between Apterygiformes *DAA2* and *DAA3* is shown above the alignment. Upstream region used for phylogenetic inference was framed with black line.

The divergence between *DAA3* and *DAA2* in Apterygiformes was driven by directional positive selection. Three sites located in the upstream region of exon 3 showed significant evidence for positive (diversifying) selection at the branch separating both gene lineages (Fig. 3). Additional nine sites showed significantly higher *dN*/*dS* ratios along the branch separating both gene lineages, compared to *dN*/*dS* ratios at the branches within gene lineages (Fig. 3). Nearly all of these sites (*n* = 8) were also located in the upstream region of exon 3 or its direct neighborhood (Fig. 3). In contrast, the analysis of selection between Apterygiformes *DAA3* and Testudines revealed only one positively selected site in the upstream region (Fig. 3).

## Discussion

Our analyses of palaeognath genomes revealed the presence of a separate lineage of MHC-IIA genes (*DAA3* in kiwis), which clustered outside the previously described avian MHC-IIA gene lineages (*DAA1* and *DAA2*). Further analyses of *DAA3* sequences showed their striking similarity to MHC-IIA sequences of nonavian reptiles (turtles). At the same time, selection inference indicated that the divergence of *DAA3* and *DAA2* lineages in kiwis was associated with a strong bout of positive selection acting on the conserved region of the membrane-proximal domain. The results suggest that *DAA3* in kiwis may constitute an ancestral MHC-IIA gene lineage in extant birds, which still retains the signal of orthology with MHC-IIA of nonavian reptiles.

Searching for orthologous relationships in avian MHC-II genes is not a trivial task. Unlike mammalian MHC-II genes, which cluster by locus and retain strong signal of orthology across highly diverged taxa (Hughes and Nei 1990), avian MHC-II has been subject to sustained concerted evolution leading to an extensive sequence homogenization between loci (Edwards et al. 1995; Westerdahl et al. 2022). However, the pioneering research by Burri et al. (2008, 2010) effectively used the conserved region of the membrane-proximal β2 domain to separate two avian MHC-IIB gene lineages (*DAB1* and *DAB2*), reflecting an ancestral duplication rather than convergence (Burri et al. 2010). Later on, the orthologous relationships of *DAB1* and *DAB2* have been traced back to the duplication event that preceded the radiation of extant birds (Goebel et al. 2017), and the same pattern has been described for avian MHC-IIA genes (Minias et al. 2023). At the same time, it has been expected that the relatively high evolutionary rate of avian MHC-IIB genes would make it difficult or impossible to identify their orthologs in nonavian amniotes (Goebel et al. 2017). In contrast, avian MHC-IIA genes are less evolutionarily dynamic (e.g. usually retain a single copy within each MHC-IIA lineage contrasting with extensive duplication of MHC-IIB genes; He et al. 2022, Minias et al. 2023), which makes them suitable to search for orthologous relationships over longer timescales. So far, analyses of MHC-IIA sequences in the chicken *Gallus gallus* suggested a putative orthology with mammalian *DR* genes, reinforcing an ancient evolutionary history of *DR*-like MHC lineage in vertebrates and a possible functional importance of the strong sequence conservation of MHC-IIA genes, even across peptide-binding residues (Salomonsen et al. 2003). Despite these early efforts to resolve orthologous relationships between avian and mammalian MHC-II genes, no evidence has been available for the orthology of MHC in birds and nonavian reptiles. Our phylogenetic analyses of MHC-IIA sequences from palaeognaths and reptiles are beginning to fill this knowledge gap.

Here, MHC-IIA sequences from kiwi (*DAA3*) and turtles formed a single well-supported (ca. 90% posterior probability) cluster, all showing relatively high nucleotide similarity (89.7%). At the same time, we found relatively low (76.6% to 81.4%) nucleotide similarity between MHC-IIA of palaeognath birds and other nonavian reptile lineages, i.e. crocodilians and squamates. As there is still no clear consensus on the amniote macroevolution, it is difficult to assess whether and how phylogenetic relationships of MHC-IIA reflect true phylogenetic relationships between major evolutionary lineages of birds and nonavian reptiles. While the lineage of lepidosauromorphs (including squamates) is generally acknowledged to have diverged in the Middle or Late Permian around 250 to 270 mya (Simões et al. 2018, Martínez et al. 2021), the origin of turtles has been particularly challenging to resolve, primarily due to conflicting morphological and molecular results (Shedlock and Edwards 2009). However, the most recent analyses suggest a close phylogenetic relationships of lineages leading to extant turtles, crocodilians, and birds, all classified within a single clade (archosauromorphs) and having diverged rapidly around the Permian–Triassic boundary (Colston et al. 2020). Hence, we conclude that the origin of *DAA3* gene lineage can be traced back to the basal archosauromorphs and its age can be estimated at ca. 250 million years.

The question on why the signature of orthology has not been retained between MHC-IIA in kiwis (*DAA3*) and crocodilians remains open, and possible scenarios may include homogenization with other MHC-II gene lineages or deletion. The same scenarios could explain the absence of *DAA3* lineage in most extant palaeognath (except Apterygiformes) and all neognath birds. In fact, biological functions of *DAA3* could have been effectively taken over by other avian gene lineages (*DAA1* and *DAA2*), possibly promoting *DAA3* pseudogenization and deletion at the early stages of radiation of extant birds. This scenario would be consistent with the birth-and-death model of MHC evolution, which is commonly evoked to explain remarkable variation in the architecture of the MHC region across vertebrates (Nei et al. 1997). Loss of functionality may have also promoted homogenization of *DAA3* with other gene lineages through gene conversion processes, where short homologous sequences are exchanged between loci (Martinsohn et al. 1999). In fact, recent comparative analysis showed that MHC gene conversion is ubiquitous in extant birds, and its rate is apparently higher at the MHC-II than MHC-I (Minias 2024). On the other hand, our phylogenetic analyses of nonconserved downstream region of MHC-IIA exon 3 revealed a clear separation of *DAA3* and *DAA2* lineages in kiwis, suggesting that *DAA3* has been particularly resistant to homogenization across the entire exon length. It cannot be, however, excluded that this resistance of *DAA3* to the homogenizing effects of gene conversion is specific to kiwis and that this gene lineage has effectively undergone homogenization in other avian orders. Finally, it is worth noting that despite the retention of *DAA3*, kiwis have apparently lost *DAA1* gene over evolutionary time. The loss of *DAA1* gene occurred independently in several other avian lineages, including Galloanserae (landfowl and waterfowl), Gruiformes, Sphenisciformes, and Accipitriformes (Minias et al. 2023). This pattern provides further support for high evolutionary lability in the structural organization of avian MHC-II region.

While it cannot be excluded that *DAA3* and *DAA2* genes have similar functionality, a strong signal of directional positive selection was detected along the phylogenetic branch separating these two genes, concentrated in the conserved region of membrane-proximal α2 domain. This region is primarily involved in interactions with the membrane-proximal β2 domain, being responsible for specific pairing and binding between the two chains of MHC-II molecule (Burri et al. 2010). Our previous analyses revealed a tight coevolution of MHC-IIA and MHC-IIB gene lineages across divergent avian clades, where phylogenetic distribution of *DAA1*/*DAA2* lineages perfectly matched the distribution of *DAB1*/*DAB2* lineages (Minias et al. 2023). This may suggest that *DAA3* lineage in kiwis may have a matching MHC-IIB (*DAB3*) lineage, together producing a specific type of MHC-II heterodimer. This expectation, however, could not be effectively investigated, as MHC-IIB genes are poorly assembled and highly fragmented in the available kiwi genomes. At the same time, our selection inference revealed only a negligible signal of directional positive selection along the branch connecting *DAA3* in kiwis and MHC-IIA in turtles. While this is likely to reflect strong functional conservation of MHC-IIA, we also tested whether the observed sequence similarity could be driven by convergent evolution. Phylogenetic analyses of nearly neutral third codon positions provided no evidence for sequence convergence, though resolution of the tree was limited. Consequently, we concluded that high similarity of MHC-IIA in kiwis and turtles most likely reflects their remarkable evolutionary conservation and retention of ancient orthologous relationships.

In conclusion, our analyses offer novel insights into the macroevolutionary history of the key vertebrate immune genes, the MHC. We provided the first evidence for orthologous relationships of MHC-IIA genes of birds and nonavian reptiles, tracing their origin to basal archosauromorphs ca. 250 mya. The study also reinforces the view that rapid accumulation of high-quality genome assemblies across divergent nonmodel species can substantially advance our understanding of gene evolution.

## Supplementary Material

evae211_Supplementary_Data

## Data Availability

GenBank numbers of all sequences used in this study are provided in the Supplementary material (supplementary tables S1 and S2, Supplementary Material online).
