# Supplementary material for: Palaeognaths Reveal Evolutionary Ancestry of the Avian Major Histocompatibility Complex Class II
Source: Genome Biol Evol. 2024 Oct 3;16(10):evae211. doi: 10.1093/gbe/evae211 (PMC11487930; doi:10.1093/gbe/evae211)
Supplement: evae211_Supplementary_Data [file evae211_supplementary_data.zip › ESM Figures S1-S2.docx]

**Electronic Supplementary Material**

**Palaeognaths reveal evolutionary ancestry of the avian Major Histocompatibility Complex class II**

**Piotr Minias ^1,^*, Wiesław Babik ^2^**

^1^University of Lodz, Faculty of Biology and Environmental Protection, Department of Biodiversity Studies and Bioeducation, Banacha 1/3, 90-237 Lodz, Poland

^2^Jagiellonian University, Institute of Environmental Sciences, Faculty of Biology, Kraków, Poland

*Corresponding author: E-mail: pminias@op.pl


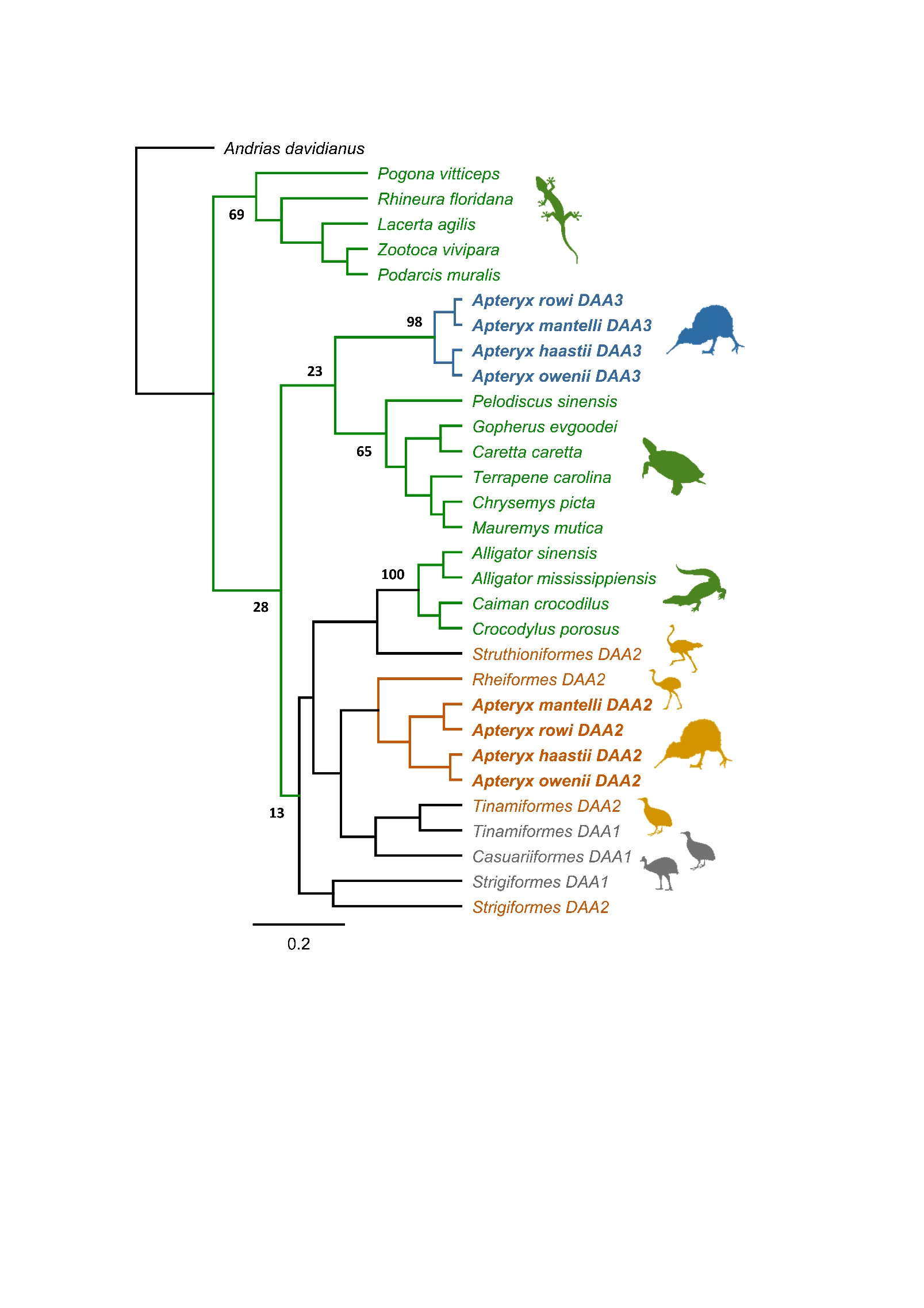
**Fig. S1.**–Consensus Bayesian topology of MHC-IIA sequences in Palaeognathae birds and reptiles. Three avian MHC-IIA gene lineages are marked in grey (*DAA1*), yellowish (*DAA2*), and blue (*DAA3*), while reptilian MHC-IIA sequences are marked in green. Kiwi (Apterygiformes) *DAA* sequences are bolded. Phylogenetic relationships were reconstructed based on the third codon positions from the upstream 90 nt region of MHC-IIA exon 3. Bayesian posterior probabilities are provided for major clusters. Owl (Strigiformes) sequences were used as *DAA1* and *DAA2* gene lineage reference (GenBank nos. BJCB01040766 and BJCB01033660; genome assembly GenBank no. GCA_004320225). *Andrias davidianus* was used as outgroup (Genbank no. KF611869). Silhouettes mark different orders of Palaeognathae birds (Apterygiformes, Casuariiformes, Rheiformes, Struthioniformes, and Tinamiformes) and reptiles (Crocodylia, Squamata, and Testudines).

**
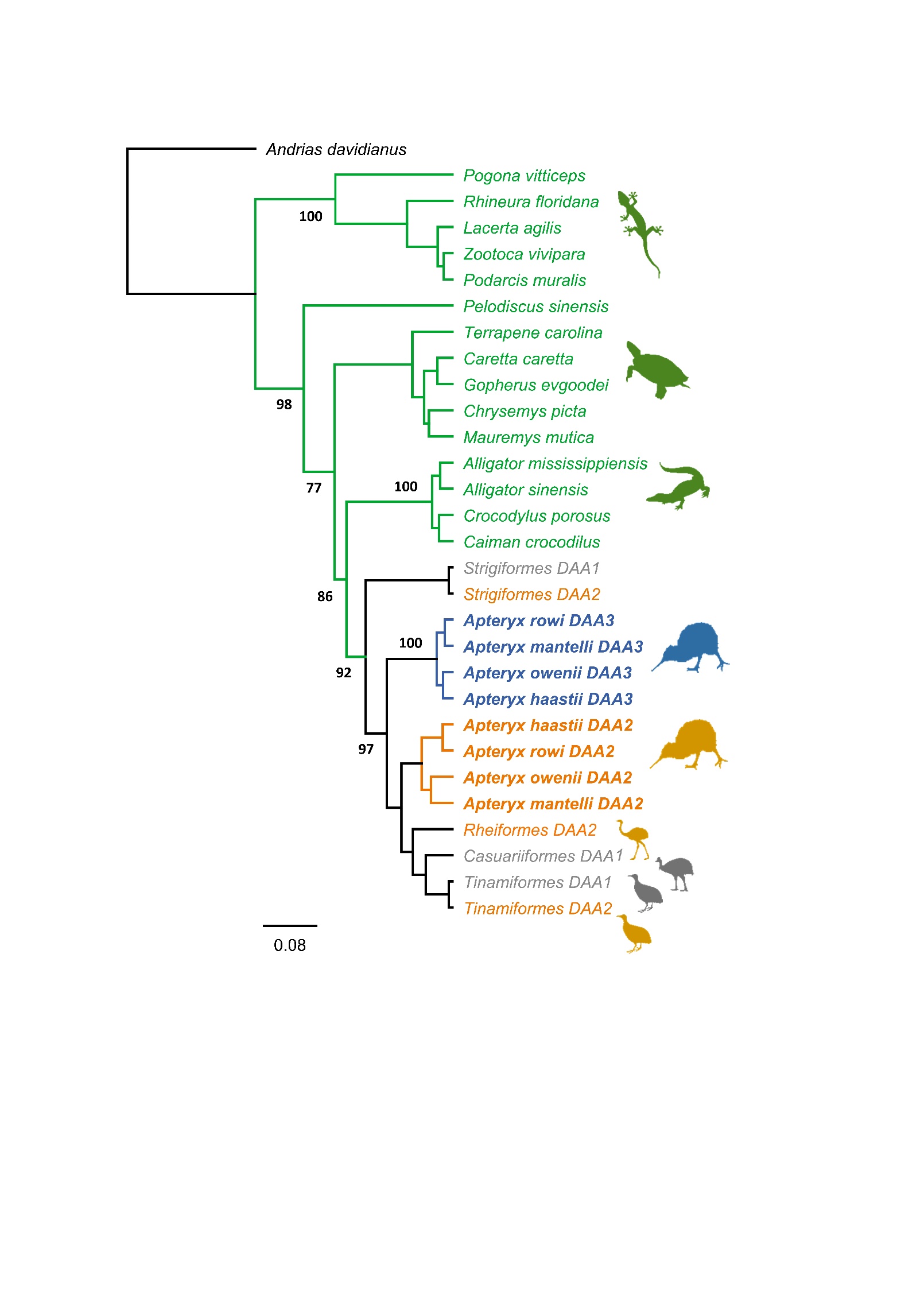
Fig. S2.**–Consensus Bayesian topology of MHC-IIA sequences in Palaeognathae birds and reptiles. Three avian MHC-IIA gene lineages are marked in grey (*DAA1*), yellowish (*DAA2*), and blue (*DAA3*), while reptilian MHC-IIA sequences are marked in green. Kiwi (Apterygiformes) *DAA* sequences are bolded. Phylogenetic relationships were reconstructed based on the downstream 192 nt region of MHC-IIA exon 3. Bayesian posterior probabilities are provided for major clusters. Owl (Strigiformes) sequences were used as *DAA1* and *DAA2* gene lineage reference (GenBank nos. BJCB01040766 and BJCB01033660; genome assembly GenBank no. GCA_004320225). *Andrias davidianus* was used as outgroup (Genbank no. KF611869). Silhouettes mark different orders of Palaeognathae birds (Apterygiformes, Casuariiformes, Rheiformes, Struthioniformes, and Tinamiformes) and reptiles (Crocodylia, Squamata, and Testudines).
